# Cognitive development after Tetralogy of Fallot repair

**DOI:** 10.1186/1749-8090-8-S1-O135

**Published:** 2013-09-11

**Authors:** M Chira, DF Ciotlaus

**Affiliations:** 1 Cardiovascular Department, Polisano Hospital, Sibiu, Romania

## Background

Psychological development, and cognitive outcome of surgically corrected patients with Tetralogy of Fallot, could be affected by the preoperative chronic cerebral hypoxia. This study highlights the higher risk of psychological impairment when the TOF surgical correction is delayed.

## Methods

This paper studies a group of 71 patients operated in Heart Institute between September 1st 2001 and July 1st 2006, all surgically corrected without prior palliations. The surgical techniques were: transannular patch (46), infundibular patch ± PA patch (17), and transatrial and transpulmonary correction (8). The patients were divided into 2 groups, operated below 1 year of age and above 1 year of age, for comparative study of results. 58 patients were followed up, performing a pediatric psychological evaluation.

## Results

The effects over patients IQ of two major components were studied: preoperative chronic hypoxia and family environment. Major differences were noticed between the two groups, as followed: the patients IQ values were significantly statistic different among the two groups (higher values for patients operated below 1 year of age), there is an inverse ratio between IQ values and preoperative hematocrit (statistically significant), the IQ values distribution is slightly different between the two groups of age, the IQ values in patients operated above 1 year of age are significantly different depending on parents scholar degrees.

## Conclusions

Delay in surgical correction, beyond 1 year of age, in patients with Tetralogy of Fallot, could have deleterious effects over cognitive outcome of these patients (longer period of preoperative chronic cerebral hypoxia). The cognitive outcome also correlates with parents scholar degrees, in older patients.

